# Diagnostic Pathology – Editorial

**DOI:** 10.1186/1746-1596-2-2

**Published:** 2007-01-10

**Authors:** Klaus Kayser

**Affiliations:** 1 Director UICC-TPCC, Institute of Pathology, Charite, Berlin, Germany

## Editorial

Dear friends, colleagues and interested scientists,

First of all I want to express my deep gratitude for your interest in our new journal *Diagnostic Pathology*, and I want to encourage you to assist us further on in promoting the journal into a high standard, innovative, and easy to handle instrument for distribution, discussion and promotion of surgical pathology and associated fields of medicine and science.

At the end of this year our open access journal *Diagnostic Pathology *does exist for about nine months. It has been designed to cover all aspects of diagnostic surgical pathology or tissue based diagnosis and related medical and biological disciplines. An editorial board of well – known experts assures the scientific quality of the submitted articles. In the beginning we had to learn how to handle the new technique of digital formalization of scientific articles and the journal too. The "formal trails" are now set up to the benefit of our authors. For example, the process of reviewing lasted for about two months in the beginning. Nowadays, most of the articles are peer reviewed within two weeks or even less to give the authors the full advantage of electronic communication. Our editorial team consists of active young experts who really enjoy their duty accompanied by internationally recognized scientists. All authors who submit articles can be assured that the scientific quality is checked completely neutral. In addition, we take into account the working conditions of our colleagues who might not have access to institutional support of high scientific standards. We do hope that we have found a balance between necessary scientific quality and the conditions to work out research results.

In order to guide our authors our journal offers a broad spectrum of article categories ranging from case reports to hypothesis, and from commentary to methodology. Articles dealing with database and debates have not been published so far. Most of the articles display case reports, which enjoy a frequent access by the readers too. For example, two of the 19 published case reports have been read by more than 1000 readers. We can conclude that case reports still contain attractive information, even in the times of data base research. The top ten most accessed articles include reviews, research and also case reports. Within a period of less than six months nearly 4000 colleagues read our most frequently accessed article on schizophrenia proteomics. All of our top ten articles had more than 900 readers each in this short period of time. Therefore, we are convinced that our idea of an open access journal addressed to diagnostic surgical pathology will have a good chance to become well established and recognized within the scenario of conventional scientific journals.

As we have gathered our experience with electronic journals in editing the first solely electronically published journal in surgical pathology (*Electronic Journal of Pathology and Histology *in 1995) we will try to include novel technologies in this journal too. The new ideas include the introduction of interactive publication, i.e., an extension of already published data by additional similar investigations not necessary done by the same authors; the access to image servers that host large images of similar cases or diagnoses; an interactive reading of scientists allowing to add statements or comments into the published article; or the performance of computer programs established by the authors and altered by readers. The implementation of these ideas will open a new area in scientific publication and allow to take advantage of electronic distribution of scientific data. In addition, it will combine routine diagnostic pathology with on-line research and quality assurance, for example by automated generation of references supporting or confirming a diagnostic statement. We will certainly encourage our publisher to proceed in the described direction having established the present performance and stage of our *Diagnostic Pathology*.

Coming back to our present stage, we are fully satisfied with the number of submitted articles and their quality. Out of a total of 68 submissions we had to reject 13 articles. We are able to publish four to eight articles per month, and we are convinced that we can even increase this rate in the near future. Unfortunately, the costs of open access publication are quite high for many authors. We try to help authors not possessing the necessary financial resources as much as possible. One of our efforts is to support colleagues working in developing countries, as we believe that science should be free of financial environment and interest as far as the circumstances permit.

Therefore, we would like to encourage all our colleagues and especially our young colleagues and those who are interested in a broad, frequently used and free access to their articles to submit their ideas, research results, overviews, or observations on individual diagnosis, patients, or disease development to us. We assure all colleagues that our fast review process will take into consideration all aspects of their work conditions as long as a certain level of scientific quality is maintained. All our reviewers have done this duty to my full satisfaction, and I want to express my deep gratitude to them.

I wish all our readers, authors, interested scientists, and members of our editorial board a Merry Christmas to personally enjoying cheerfulness, freedom, and peace, as well as a Happy, Healthy and Prosperous New Year 2007.

Berlin, December 2006

Klaus Kayser M.D., Ph.D.

Editor in Chief.

